# Normal mammary gland development after MMTV-Cre mediated conditional PAK4 gene depletion

**DOI:** 10.1038/s41598-019-50819-4

**Published:** 2019-10-08

**Authors:** Parisa Rabieifar, Ting Zhuang, Tânia D. F. Costa, Miao Zhao, Staffan Strömblad

**Affiliations:** 1 Department of Biosciences and Nutrition, Karolinska Institutet, Stockholm, Sweden; 2 Present Address: Henan Collaborative Innovation Center of Molecular Diagnosis and Laboratory Medicine, School of Laboratory Medicine, Xinxiang Medical University, Xinxiang, Henan Province P.R. China; 3 Present Address: Department of Immunology, Genetics and Pathology, Neuro-Oncology, Uppsala University, Uppsala, Sweden

**Keywords:** Breast cancer, Cell proliferation

## Abstract

p21-activated kinases (PAKs) are serine/threonine kinases functioning as downstream effectors of the small GTPases Rac1 and Cdc42. Members of the PAK family are overexpressed in human breast cancer, but their role in mammary gland development is not fully explored. Here we examined the functional role of PAK4 in mammary gland development by creating a mouse model of MMTV-Cre driven conditional PAK4 gene depletion in the mammary gland. The PAK4 conditional knock-out mice were born healthy, with no observed developmental deficits. Mammary gland whole-mounts revealed no defects in ductal formation or elongation of the mammary tree through the fat pad. PAK4 gene depletion also did not alter proliferation and invasion of the mammary epithelium in young virgin mice. Moreover, adult mice gave birth to healthy pups with normal body weight upon weaning. This implies that MMTV-Cre induced gene depletion of PAK4 in mice does not impair normal mammary gland development and thereby provides an *in vivo* model that can be explored for examination of the potential function of PAK4 in breast cancer.

## Introduction

The mammary gland is a highly ductal organ mainly composed of two distinct cell compartments, the epithelium, and the surrounding stroma, which are derived from ectoderm and mesoderm during embryogenesis^1,2^. Unlike most other epithelial organs, development of the mammary gland occurs postnatally^3,4^. Many signaling molecules such as growth hormone, estrogen, and growth factors stimulate formation and invasion of terminal end buds (TEB) to the mammary fat pad by regulating extracellular matrix (ECM) proteins^4–6^.

Within the ECM, integrins act as chemomechanical sensors of mammary epithelial cells, which thereby receive signals from the surrounding environment and regulate cell proliferation, polarization, and further morphological organization through its downstream mediators such as the Rho family small GTPases^7,8^.

The p21-activated kinase (PAK) family of serine/threonine protein kinases are downstream effectors of the small Rho family GTPases Cdc42 and Rac^9^. PAKs are categorized into two subgroups based on their sequence homology. Group I includes PAK1, 2 and 3, while the Group II includes PAK4, 5 and 6.

PAKs control major subcellular activities, such as cytoskeletal remodeling, mitotic progression and DNA damage response and play essential roles in organ formation throughout mammalian development^10^. Moreover, their overexpression is associated with cell proliferation, cell survival, invasion, angiogenesis and epithelial-mesenchymal transition (EMT), which are connected with cancer initiation and progression^11^.

PAK4, a member of group II PAKs is expressed both during development and in adult tissues^11–13^. PAK4 is involved in a variety of cytoskeletal regulation such as promoting filopodia formation, dissolution of stress fibers, controlling actin polymerization and depolymerization as well as focal adhesion turnover^12–15^. Consistently, PAK4 overexpression is correlated with poor patient outcome in breast cancer patients, and its overexpression in cancer cell lines was shown to increase cell survival, anchorage-independent growth, cell migration, and invasion^12,13,16–18^. PAK4 also plays an essential role during embryonic development, as complete depletion of PAK4 in mice caused embryonic lethality with severe defects in the heart, brain, and vasculature of the animals^19^; However, the role of PAK4 in mammary gland development has not been investigated. Due to the early embryonic lethality of conventional PAK4 knock-out mice, conditional PAK4 knock-out mice have been developed to study its role in different tissue’s development^20–22^. To this end, we created a transgenic mouse model to conditionally deplete PAK4 in the mammary gland by crossing MMTV-Cre mice with PAK4^floxed/floxed^ mice. We observed that PAK4 conditional knock-out mice were viable, produced standard litter sizes, and pups with normal body weight upon weaning. Moreover, PAK4 knock-out mice exhibited healthy ductal morphology. These results indicate that PAK4 is dispensable for mouse mammary gland development.

## Results

### MMTV-Cre mediated conditional disruption of PAK4 in the mouse mammary gland

**Figure 1:**
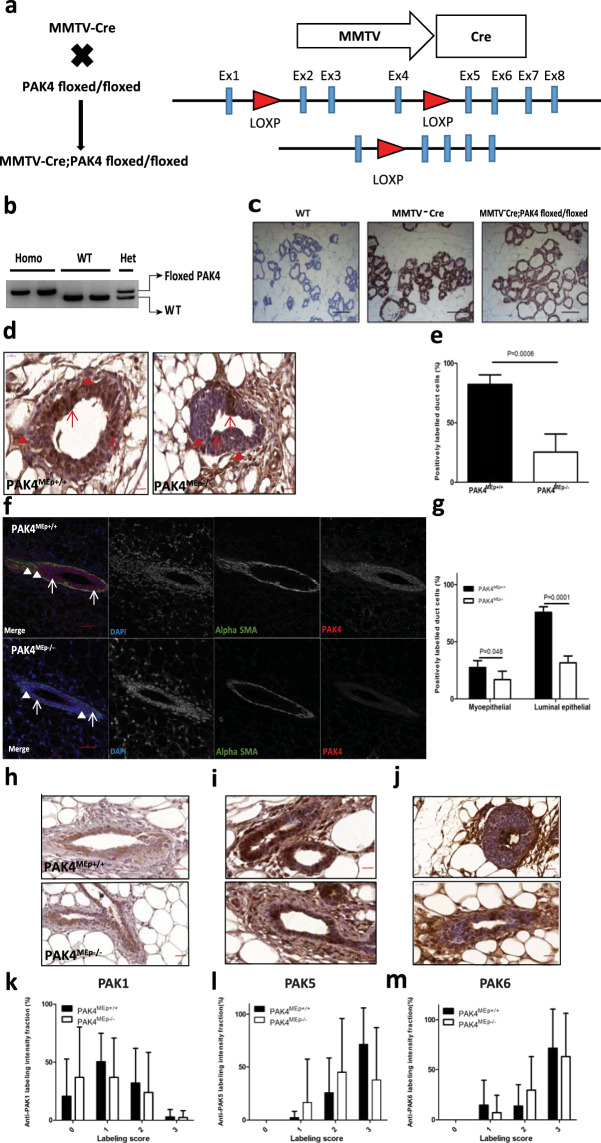
Conditional gene depletion of PAK4 in the mammary epithelium using the Cre-Lox system. (**a)** Schematic representation of the strategy for the generation of a mouse model with conditional PAK4 gene depletion in the mammary epithelium. Exons are indicated by light blue rectangles, and Lox P sites are indicated by red triangles. As previously described, Sox2-Cre expression and consequent recombination of LOXP sites result in the depletion of exons 2–4 in the mouse PAK4 gene^20^. **(b)** Inverted image of PCR analysis of genomic DNA from 21- day old mice. The upper band displays the floxed PAK4 allele, while the lower band displays a PAK4 WT allele. Thus, the appearance of the upper band alone displays homozygous floxed PAK4 allele; the lower band alone represents WT PAK4 allele; while the presence of both bands means that the mouse is PAK4 heterozygous, i.e., one WT and one floxed allele. Full gel is shown in Supplementary Fig. S1a. **(c)** Immunohistochemistry labeling of Cre in thoracic mammary glands isolated from mice of different genotypes at Lactation day 2. Scale bar: 100 μm. **(d)** Representative immunohistochemistry labeling with an anti-PAK4 pab of inguinal mammary glands from PAK4^MEp+/+^ and PAK4^MEp−/−^ mice in virgin week 4 mice with hematoxylin nuclear counterstaining. Scale bar is 20 μm. Arrows indicate positively and arrowheads indicate negatively labelled cells. **(e)** Quantification of the fraction of positively PAK4 labelled duct cells. Means represent quantification of positively labelled cells in 3 to 7 evenly distributed, similar-sized ducts each of 5 mice for each group (n = 5; p = 0.0006 according to an unpaired t-test). **(f)** Representative immunofluorescence labelling of PAK4 (Red) and the myoepithelial marker α-SMA (Green) of inguinal mammary glands from PAK4^MEp+/+^ and PAK4^MEp−/−^ mice in virgin week 4 mice with Hoechst nuclear counterstaining in blue. Scale bar is 50 µm. Arrows indicate positive, and arrowheads indicate negative labelled cells. **(g)** Quantification of the fraction of cells positively labelled for PAK4 among the luminal and myoepithelial cells, respectively. Means represent quantification of positively labelled cells in 3 to 7 evenly distributed, similar-sized ducts each of 5 mice for each group. Shown values represent mean ± s.d. and p-values were calculated by an unpaired t-test: myoepithelial (P = 0.048, n = 5) and luminal epithelial (P = 0.0001, n = 5). Arrows indicate positive and arrowheads indicate negative labeling. **(h**–**j)** Representative immunohistochemistry images of labeling with anti-PAK1 **(h)**, anti-PAK5 **(i)**, and anti-PAK6 **(j)** antibodies of sections from inguinal mammary glands from PAK4^MEp+/+^ and PAK4^MEp−/−^ virgin week 4 mice with hematoxylin nuclear counterstaining. Scale bar is 20 μm. **(k–m)** Anti-PAK1 **(k)**, anti-PAK5 **(l)** and anti-PAK6 **(m)** antibody labelling intensity was graded according to the level of staining intensity (0 = Negative; 1 = weakly positive; 2 = Moderately positive; 3 = Strongly positive). Quantification was performed in 3 to 7 evenly distributed, similar-sized ducts each from 5 different mice for each group (n = 5).

Given that complete depletion of the PAK4 gene in the mouse causes embryonic lethality^19^, we created a mouse model to deplete PAK4 in the mammary epithelium using the Cre/loxP system; by crossing MMTV-Cre mice with PAK4-floxed mice^20,23,24^ (Fig. 1a) MMTV-Cre mice (line D) have been used in breeding, as this line has minimal effects on mammary gland development compared to other lines^23,25^. The MMTV-Cre mice were used as a control group in this study (Fig. 1a). PAK4 flox was genotyped by PCR analysis of genomic DNA, identifying wild-type mice, as well as homozygous and heterozygous PAK4 knock-out mice (Fig. 1b). When MMTV-Cre; PAK4^fl/+^ mice were crossed with PAK4^fl/fl^ mice, four different genotypes were born approximately at the expected Mendelian ratio, i.e. Wild-type (PAK^fl/fl^ and PAK4^fl/+^) 49%; Het (MMTV-Cre; PAK4^fl/+^) 23%; and Homo PAK4 KO (MMTV-Cre; PAK4^fl/fl^) 28% (Table 1). Hereafter, for simplicity, mice with the MMTV-Cre; PAK4^fl/fl^ genotype will be referred to as PAK4^MEp−/−^ and MMTV-Cre mice as PAK4^MEp+/+^. Within the same genotypes, female and male displayed an approximately equal distribution; suggesting that loss of PAK4 in the mammary epithelium does not affect survival in any of the sexes.

**Table 1:** Genotypes of the progeny.

Total	PAK4 ^fl/fl^	Pak4 ^fl/+^	MMTV-Cre;PAK4 ^fl/+^	MMTV-Cre;PAK4 ^fl/fl^
137	34 (25%)	33 (24%)	31 (23%)	39 (28%)

Genotypes of progeny produced by crossing MMTV-Cre; PAK4fl/+ mice with PAK4fl/fl mice.

The number of mice from each genotype is indicated as the percentage of mice falling into each genotype within brackets.

To test the efficiency of the PAK4 gene depletion in PAK4^MEp−/−^ mouse mammary glands, MMTV-Cre expression pattern was examined as an indicator of PAK4 depletion. Mosaicism of Cre expression pattern in the MMTV-Cre mice model has been reported^23,25,26^. Based on estimation in both PAK4^MEp+/+^ mice and PAK4^MEp−/−^ mice, more than 90% of the mammary epithelial cells displayed Cre positive staining, and the Cre staining patterns were similar in the two groups (Fig. 1c). Samples from wild-type mice without Cre expression was used here as a negative control. Considering intrinsic limitations of the Cre/LoxP model^23,24,25^, we then performed immunohistochemistry (IHC) using an anti-PAK4 pab and quantified positively labelled cells in the whole mammary duct, displaying a strong reduction of anti-PAK4 labeling in the mammary glands of PAK4^MEp−/−^ mice (Fig. 1d–e). Consistently, immunofluorescent labeling using the same anti-PAK4 pab together with labeling of the myoepithelial marker alpha smooth muscle actin (α-SMA) showed significantly lower anti-PAK4 labeling of both luminal and myoepithelial cells in the PAK4^MEp−/−^ mice compared to PAK4^MEp+/+^ mice (Fig. 1f,g). However, there was still a notable fraction of duct cells in the PAK4^MEp−/−^ mice labeling positive with the anti-PAK4 pab. Thus, the MMTV-Cre mediated recombination was not complete, leading to mosaic expression, consistent with what was previously reported for the MMTV-Cre model^23,25,26^. Nevertheless, these data suggest that our PAK4 conditional knock-out mice have an efficient PAK4 depletion in the vast majority of the mammary epithelial cells.

Considering the possibility that upregulation of other members of the PAK family might compensate for the absence of PAK4, we performed IHC using antibodies against PAK1^27,28^, PAK5, and PAK6. However, we did not detect any increased mammary gland labelling for any of these antibodies in the absence of PAK4 as compared to control glands (Fig. 1h–m).

### PAK4 gene depletion in the mammary gland does not alter ductal elongation and branching

**Figure 2:**
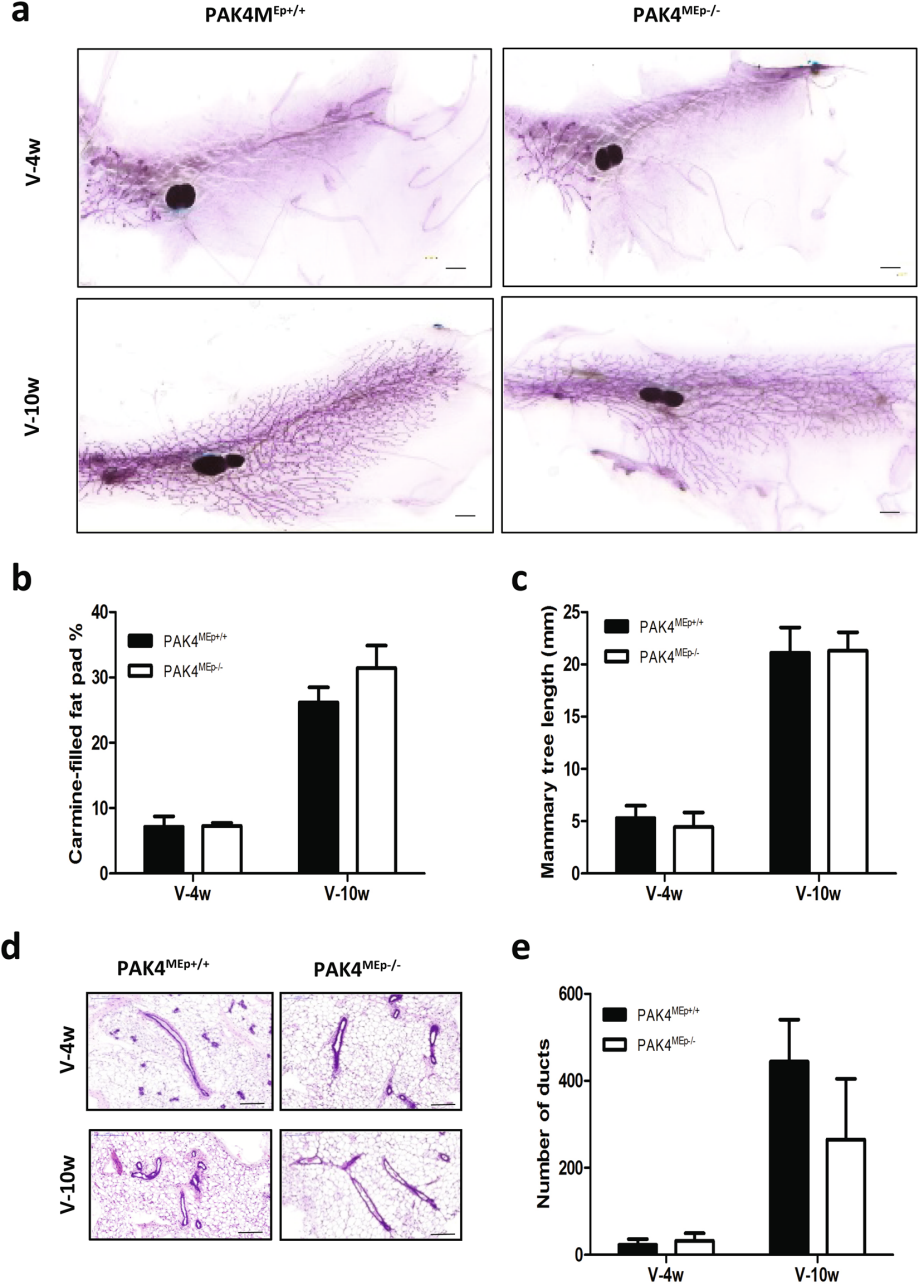
MMTV-Cre-driven PAK4 gene depletion does not impair mammary gland development. **(a)** Representative whole-mount staining of mammary glands obtained from PAK4^MEp−/−^ and PAK4^MEp+/+^ mice during the virgin week 4 and week 10 developmental stages. Scale bar is 1 mm **(b)** Whole-mount staining quantification of carmine-filled fat-pad (%) from PAK4^MEp+/+^ and PAK4^MEp−/−^ mice at virgin week 4 (P = 0.8, n = 6) and week 10 (P = 0.07, n = 6) stages of development. **(c)** Mammary duct length measurement in whole-mounts staining from PAK4^MEp+/+^ and PAK4^MEp−/−^ mice at week 4 (P = 0.2, n = 6) and week 10 (P = 0.8, n = 6) of mouse development. **(d)** Representative H&E staining of mammary glands from PAK4^MEp+/+^ and PAK4^MEp−/−^ mice at week 4 (n = 6) and week 10 (n = 6) of mouse development. Scale bar is 200 μm. **(e)** Quantification of the number of ducts in H&E stained sections of mammary glands from PAK4^MEp−/−^ and PAK4^MEp+/+^ mice at week 4 (P = 0.9, n = 6) and week 10 (P = 0.09, n = 6) of mouse development. Shown values represent mean ± s.d. and p-values were derived from unpaired t-tests.

To examine the ductal growth within the mammary gland in juvenile virgin and adult virgin mice, mammary glands were isolated from mice at 4 and 10 weeks of age. To determine the potential effect of PAK4 depletion on the morphogenesis of the mammary gland, whole-mount preparations were used for measurement of the area fraction covered by mammary epithelium in the entire mammary fat pad. We found that the mammary fat pads were filled by epithelial tissue in the PAK4^MEp−/−^ mice to the same extent as in their aged-matched PAK4^MEp+/+^ mice (Fig. 2a,b). We further performed a quantitative analysis of mammary tree elongation along the fat pad by measuring mammary tree length from the nipple. Quantitative analysis showed that the relative duct length in whole mounts from PAK4^MEp−/−^ mice was similar to PAK4^MEp+/+^ mice at both 4 and 10 weeks of age (Fig. 2c). To explore potential subtle differences in the ductal structure, we analyzed ductal structures in hematoxylin and eosin (H&E) staining of tissue sections. This staining revealed that the ductal structures in PAK4^MEp−/−^ mammary glands were evenly distributed along the fat cells and were not distinguishable from those of PAK4^MEp+/+^ mice (Fig. 2d). Moreover, the number of mammary ducts was similar between the two groups (Fig. 2e).

### Loss of PAK4 does not alter cell proliferation or invasion marker expression in the mammary epithelium

**Figure 3:**
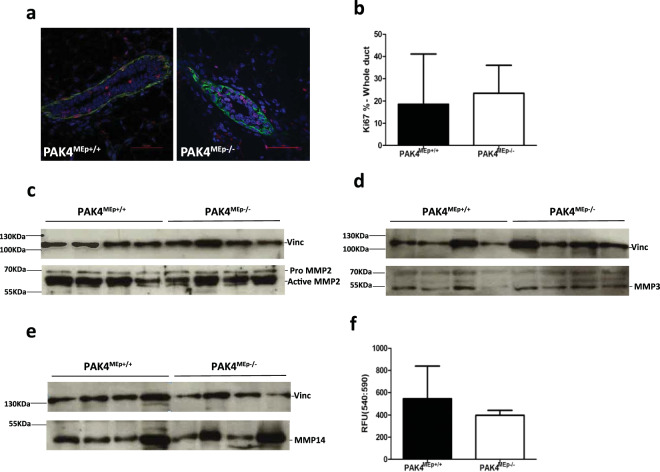
PAK4 depletion does not interfere with normal mammary epithelium proliferation. **(a)** Representative immunofluorescence labelling of Ki67 (Red) and α-SMA (Green) in inguinal mammary glands from PAK4^MEp+/+^ and PAK4^MEp−/−^ mice in virgin week 4 mice with DAPI nuclear counterstaining. **(b)** Quantification of the percentage of Ki67 positive cells in the mammary gland duct, 10 ducts from each gland were imaged and quantified. Shown values represent mean ± s.d. and p-value calculated by an unpaired t-test (P = 0.6, n = 6). **(c–e)** Immunoblot analysis of four mammary gland whole cell lysates each from virgin week 4 PAK4^MEp+/+^ and PAK4^MEp−/−^ mice of MMP2, MMP3, and MMP14. Immunoblotting for Vinculin (Vinc) was used as a loading control. The position of size markers are shown to the left and the assumed identities of the bands of interest indicated to the right. Full images  are showed in Supplementary Fig. S1b–d. **(f)** Total MMP activity was measured in 100 μg total protein from four weeks old virgin mammary glands. Shown values represent mean ± s.d. and a p-value calculated by an unpaired t-test (P = 0.3, n = 4).

Considering the known role of PAK4 in cell proliferation and invasion^29–31^, we next sought to determine the status of cell proliferation and expression of known markers for invasion within the mammary duct of virgin week 4 mice, a time point where the mammary epithelium is highly proliferative. To quantify cell proliferation within the duct, we labeled tissues for Ki67 and consistent with our previous results, lack of PAK4 did not alter cell proliferation in PAK4^MEp−/−^ mice (Fig. 3a,b).

Next, we measured matrix metalloproteinases (MMPs) expression level, since MMPs are principal executors of matrix remodeling during mammary gland development^32,33^. Mammary gland tissue lysates from PAK4^MEp+/+^ and PAK4^MEp−/−^ mice showed similar expression levels of MMP2, MMP3, and MMP14, suggesting no expression differences among these key invasion markers (Fig. 3c–e). Consistently, we also did not detect any difference in total MMP activity (Fig. 3f).

### PAK4 knock-out mothers were able to nourish pups sufficiently

**Figure 4:**
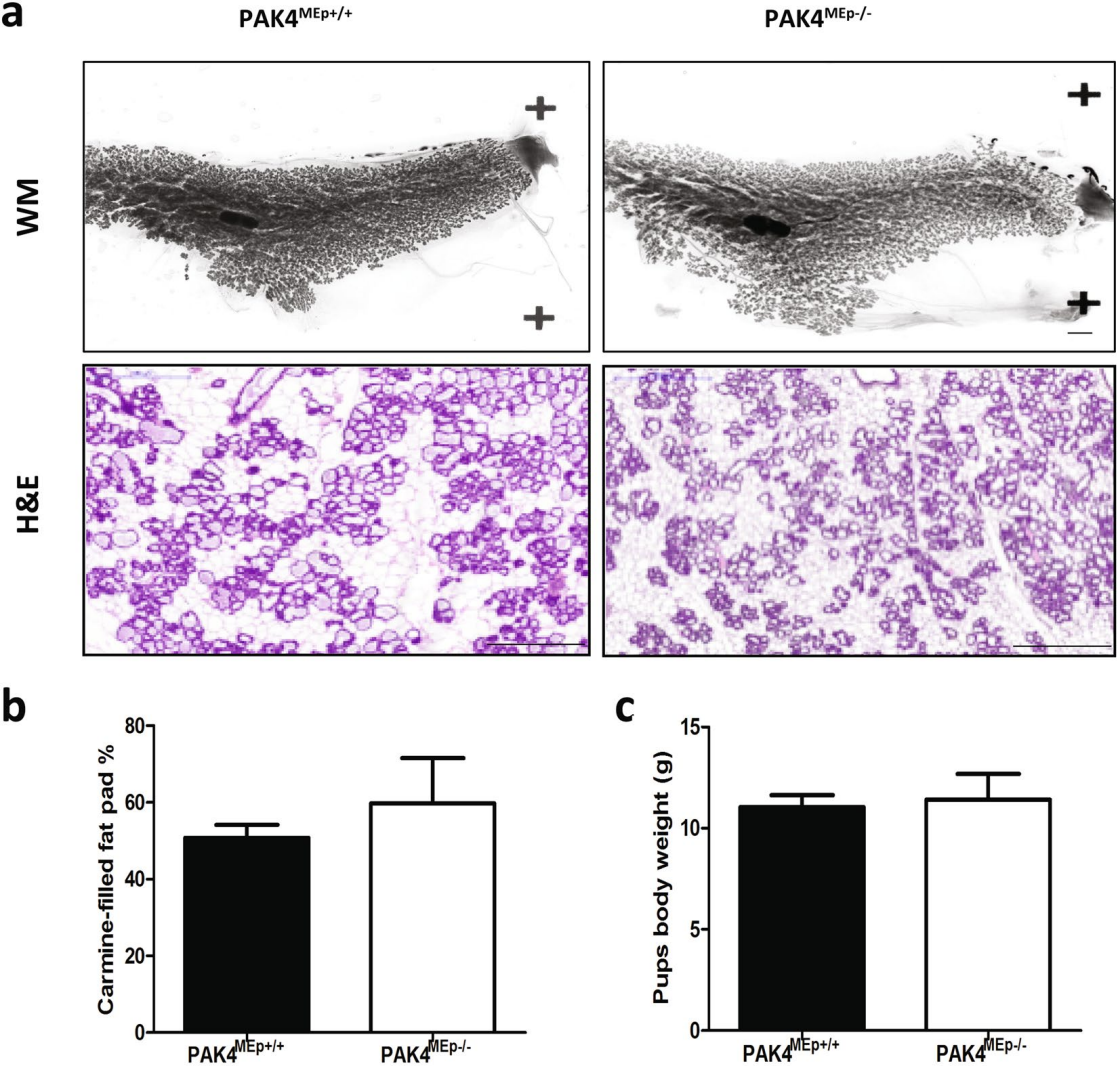
PAK4 depletion does not impair mammary gland lactation. **(a)** Representative whole-mounts (WM) and H&E staining of mammary glands obtained from PAK4^MEp+/+^ and PAK4^MEp−/−^ mice at lactation day 2 stage. Scale bar is 200 μm. **(b)** Whole-mount staining quantification of carmine-filled fat-pad (%) from PAK4^MEp+/+^ and PAK4^MEp−/−^mice at Lactation day 2 (P = 0.4, n = 4). **(c)** Average body weights of pups from PAK4^MEp+/+^ and PAK4^MEp−/−^ mothers when weaning 21 days after birth (P = 0.5, n = 6). Values represent mean ± s.d. and p-values were derived from unpaired t-tests.

We first examined and compared the overall structure of the mammary glands from PAK4 knock-out and wild-type females. In carmine red stained whole-mounts from PAK4^MEp+/+^ and PAK4^MEp−/−^ mothers displayed indistinguishable ductal epithelial development at lactation day 2, and we could not detect any difference in the percentage of epithelial cells coverage of carmine filled fat pads. Moreover, the alveolar units of PAK4^MEp−/−^ females were as fully developed as in PAK4^MEp+/+^ females (Fig. 4a,b). Both groups produced standard litter sizes (Table 1) and similar pup body weight upon weaning (Fig. 4c). Together, this indicates that MMTV-Cre driven conditional gene depletion of PAK4 caused no defects in mammary gland development or function.

## Discussion

Using MMTV-Cre mediated PAK4 depletion in mammary gland epithelium, we have shown that targeted inactivation of PAK4 in mammary epithelial cells does not impair mammary gland development; this suggests that PAK4 is dispensable for murine mammary gland development and function.

PAK4 is ubiquitously expressed throughout embryonic development and in adult tissues^19,34^. Transgenic mice with constitutive PAK4 gene depletion do not survive past embryonic day 11.5^19^; therefore conditional gene depletion strategies have been developed in the field to study its role in different organs development^20–22,35^. Conditional depletion of PAK4 in heart, vasculature, and brain caused severe defects during embryonic development while PAK4 depletion in the pancreas did not cause any evident defect in the pancreatic tissue development and function^20–22,35^. However, the potential function of PAK4 in mammary gland development has remained unclear. To this end, we found that the mammary tree development upon PAK4 depletion was neither affected during pre-puberty nor in young adult virgin female mice; moreover PAK4 knock-out mice gave birth to healthy progeny and were able to nurse them as well as the control mice. However, it is possible that mammary gland development is dependent on other members of the PAK family, since lack of PAK1 in the mammary gland impaired lobuloalveolar development and cell differentiation^36^, while conventional depletion of PAK5 and PAK6 resulted in normal mammary gland development^37,38^. This supports the idea that different members of the PAK family fulfill different functions throughout organ development^34^.

PAK4 is overexpressed in breast cancer cell lines as well as in breast cancer patients, and its overexpression is accompanied by poor patient outcome^11,16,29,39^. However, our understanding of the *in vivo* function of PAK4 in breast cancer remains limited. Given that our model for conditional PAK4 gene depletion in the mouse mammary gland displays no apparent defect in organ development and function, this can serve as a useful model to study the *in vivo* role of PAK4 in breast cancer through crossing these mice with mammary tumor models such as MMTV-PyMT and MMTV-Her2^40–42^. In fact, we recently crossed the here presented mouse model of MMTV-Cre driven conditional PAK4 gene depletion with the MMTV-PyMT breast cancer model and observed an increased mammary tumor latency upon PAK4 depletion^39^.

Nevertheless, one should also be aware of the Cre-mosaicism that we observed, consistent with previous reports upon the use of  MMTV-Cre and similar models^23–26^, which could complicate their use in an evolutionary disease like cancer, with the possibility of a selection of cells in which the gene of interest remains expressed^39,43^. Using a reporter gene could be a useful approach to overcome this problem in future studies^44^.

In summary, our data suggest that lack of PAK4 does not alter normal mammary gland development. Therefore, our mouse model of conditional depletion of PAK4 in the mammary epithelium can be useful for testing potential *in vivo* functions of PAK4 in mammary physiology and diseases such as cancer.

## Materials and Methods

### Animals

All the experimental procedures performed on animals in this study have been performed in accordance with Swedish and European Union guidelines and approved by Stockholm South and Linköping Animal Ethics Committees. To avoid the influence of social isolation, animals were housed in groups with 12:12 light: dark cycle, controlled humidity (55% ± 5%), controlled temperature (21 °C ± 2 °C) and free access to food and water.

In order to generate PAK4^MEp−/−^ mice, PAK4^fl/fl^ mice (B6.129S2(FVB)-Pak4^tm2.1Amin^/J, a gift from Audrey Minden)^20^ were crossed with MMTV-Cre/Line D mice (Tg(MMTV-cre)4Mam/J, Jackson Laboratory)^23,25^. All mice have been maintained on a B6 background. For conditional gene depletion in the mammary gland, PAK4^fl/fl^ mice were first crossed with MMTV-Cre mice to generate MMTV-Cre; PAK4^fl/+^. Such animals were then crossed with PAK4^fl/fl^ mice, resulting in littermates with PAK^fl/fl^, PAK4^fl/+^, MMTV-Cre; PAK4^fl/+^ and MMTV-Cre; PAK4^fl/fl^ genotypes (Table 1).

Genomic DNA was prepared from biopsies using the fast tissue-to-PCR kit (#K1091, Fermentas). Mice were genotyped for heterozygous and homozygous knock-out of PAK4 according to Tian *et al*^20^. The primer pairs used (synthesized by ThermoFisher) were as follows: Pak4 flox: F, 5′-CGGATATTGTCACCCACACCAG-3′ and R, 5′-CTAACAGGGACAGGAGCT-3′. DNA band was visualized on 2% agar gels stained with GelRed (41003, Biotium). All mammary gland tissues used in this paper are from female mice.

### Tissue Collection

Mice were sacrificed by cervical dislocation after anesthesia with isoflurane, and the mammary glands were collected. #1 and #2 thoracic mammary glands were quickly frozen and accordingly used for RNA and protein extraction. The #10 inguinal mammary gland was dissected, flattened on a piece of paper, fixed in 4% Paraformaldehyde overnight, then washed with PBS and kept in 70% ethanol for paraffin embedding and later used for immunohistochemistry.

### Whole-mount staining of mammary glands

The #4 inguinal mammary gland was collected to determine the area where mammary glands were developed in fat pads. Briefly, the samples were fixed overnight with Carnoy’s fixative (100% ethanol/chloroform/glacial acetic acid, 6:3:1). Then samples were hydrated by sequential treatment in 70%; 50%; 30%; and 10% ethanol for 15 min each. After the hydration process, samples were washed in tap water for 5 minutes and placed O/N in staining solution at RT. The staining solution was prepared by dissolving 1 g carmine (C1022, Sigma) and 2.5 g aluminum potassium sulfate (A7167, Sigma) in 500 ml water followed by boiling for 20 min. The samples were then dehydrated by sequential treatment in 70%; 95%; and 100% ethanol for 15 min each, followed by storage in xylene (28975, VWR) until scanning. Scanned images were analyzed using ImageJ/Fiji (Version 1,52i) (National Institutes of Health, NIH). Whole-mount images were cropped to only include #4 inguinal mammary gland then converted to an 8-bit image and sharpened. Area of fat pad occupied by mammary epithelium (carmine staining) was measured by adjustment of threshold automatically and demonstrated as a percentage of the total mammary fat pad occupied by the mammary epithelium.

Quantification of mammary tree branching was done on whole-mount images by measurement of the mammary tree length from the nipple to the last branch in millimeters using ImageJ/Fiji (Version 1,52i) (National Institutes of Health, NIH).

### H&E staining and immunostaining

Paraffin blocks were cut into 4 μm sections. For H&E staining, sections were stained with hematoxylin and eosin according to a standard protocol^45^. For immunohistochemistry, sections were deparaffinized by incubation in 60 °C incubator for 1 h followed by rehydration steps through washing in xylene and graded ethanol to distilled water. Samples were boiled for 20 min in antigen retrieval solution 0.01 M sodium citrate buffer (100813 M, BDH) (pH 6.0) in water. Endogenous peroxidase activity was blocked via treatment with 3% hydrogen peroxide in water (H1009, Sigma). PBS diluted normal serum from the same host species as the secondary antibody was used as a blocking buffer.

For immunohistochemistry, slides were incubated at 4 °C overnight in a humidified chamber with following antibodies α-Cre (1:100, PRB-106P, Covance), α-PAK1 (1:200, #2602, Cell signaling), α-PAK4 (1:100, 14685-1-AP, Proteintech), α-PAK5 (1:200, ab110069, Abcam), and α-PAK6 (1:200, ab37749, Abcam) diluted in blocking buffer. After three times PBS washing, slides were incubated at RT for 1 h with (1:400, Biotin α-Rabbit IgG, Jackson) followed by three washes with PBS and then were incubated RT 20 min with HRP-conjugated Streptavidin (016-030-084, Jackson). Following three washes with PBS, development was performed with diaminobenzidine (DAB) (K3467, DAKO Sweden). Then slides were counterstained with Mayer HTX staining solutions (01820, Histolab), dehydrated and mounted using DPX mounting media (44581, Sigma). Images were acquired on Panaromic MIDI II from 3DHISTECH slide scanner. All images were randomized and visually quantified in a blinded manner. Scanned slides with anti-PAK4 antibody were quantified for the fractions of positively labelled cells within the mammary ducts. Labeling intensity in the mammary ducts of PAK1, PAK5, and PAK6 antibodies were determined by grading using a four grade scoring system (0 = Negative; 1 = Weakly positive; 2 = Moderately positive; 3 = Strongly positive).

For Ki67 immunofluorescent co-labelling experiment, sections were incubated at 4 °C ON in a humidified chamber with Rabbit α-Ki67 (1:125, #12202, Cell signaling) co-labelled with Mouse α-SMA-FITC conjugated (1:300, F3777, Sigma) and for PAK4, SMA immunofluorescent co-staining, Rabbit α-PAK4 (1:100, 14685-1-AP, Proteintech) were co-labelled with Mouse α-SMA-FITC conjugated (1:300, F3777, Sigma). After washing, slides were incubated at RT for 1 h with Biotin α-Rabbit IgG (1:400, Jackson) to improve the signal for α-Ki67 and α-PAK4 labelings. Samples were then washed and incubated RT 30 min with Cy3-conjugated Streptavidin (AB_2337244, Jackson). Sections were further counterstained with Hoechst 33342 (1:1000, 14533, Sigma).

Images of α-Ki67 labeling were acquired using a Nikon A1R confocal microscope with a Plan Apo VC 60×/1.4 NA oil objective and NIS-Elements software (Nikon). Proliferation index was calculated as the percentage of Ki67 positive cells relative to the total number of the cells within the ducts.

Images of α-PAK4 labeling were acquired for quantification on a Panaromic MIDI II slide scanner from 3DHISTECH and for display by a Nikon A1R confocal microscope as described above. PAK4 positive labeling was calculated as the percentage of cells positively labelled by the α-PAK4 Pab relative to luminal epithelial and myoepithelial cells, respectively. Myoepithelial cells were defined as the cells labeling positive for α-SMA, while remaining ductal cells were considered to be epithelial cells. Image analysis was performed by counting positively labelled cells in 3 to 7 evenly distributed, similar-sized ducts each of 5 mice for each group. For all image quantifications, images were randomized and visually quantified in a blinded manner.

### Immunoblotting

Equal amounts of denatured protein were subjected to 10% SDS-PAGE (SDS-polyacrylamide gel electrophoresis), including molecular weight markers (PageRuler, #26617, Fermentas and PageRuler Plus, #266201, Fermentas) to estimate the molecular weight sizes of appearing bands, and transferred to PVDF membranes (IPVH00010, Millipore). Membranes were washed in TBST buffer (TBS containing 0.1% Tween-20), and non-specific binding sites were blocked by immersing the membranes in blocking buffer containing 5% non-fat milk (70166, Sigma) in TBST buffer for 1 h on a shaker at room temperature or overnight at 4 °C. Membranes were first probed with the following primary antibodies: α-MMP2 (1:1000, sc-10736, Santa cruz), α-MMP3 (1:1000, ab52915, Abcam) and α-MMP14 (1:1000, ab3644, Abcam). After the first blotting, an anti-vinculin antibody (1:50000, V9131, Sigma) was used to control for protein loading. Next, a peroxidase-conjugated α-rabbit (111-035-144, Jackson ImmunoResearch) secondary antibody was used. Bound antibodies were visualized with the Pierce Enhanced Chemiluminescence (ECL) Plus Western Blotting Substrate detection system (32132, ThermoFisher) according to the manufacturer’s instructions.

### MMP activity assay

The total MMP activity was measured fluorometrically using an MMP activity assay kit (ab112147, Abcam). Samples collected from inguinal mammary glands of 4 weeks old virgin mice were homogenized in 500 μl Ripa buffer. A black 96-well plate with clear bottom was used to carry out the assay. The enzymatic reaction was performed according to the manufacturer protocol by adding 50 μl of the homogenate to each well, with pre-incubation for 15 minutes. 50 μl of MMP Red Substrate solution was then added to each well. After 1 hour of incubation at 37 °C, fluorescence (relative fluorescence units, RFUs) was measured at 540 nm excitation and 590 nm emissions using a Gemini XPS microplate spectrofluorometer. Each homogenate was analyzed in triplicate. Three substrate control wells were used as negative controls, whose mean value determined the baseline that was subtracted from the sample wells.

### Statistics

A two-tailed unpaired t-test was utilized for statistical analyses. P < 0.05 was considered to represent statistical discernibility of differences.

## Supplementary information

Supplementary information

## Data Availability

Underlying data could be obtained from the corresponding author upon reasonable request.

## References

[1] Williams JMDC (1983). Mammary ductal elongation: differentiation of myoepithelium and basal lamina during branching morphogenesis. Dev Biol.

[2] Cheryll Tickle, H.-S. J. Embryonic Mammary Gland Development. *eLS* (2016).

[3] Macias H, Hinck L (2012). Mammary gland development. Wiley Interdiscip Rev Dev Biol.

[4] Inman JL, Robertson C, Mott JD, Bissell MJ (2015). Mammary gland development: cell fate specification, stem cells and the microenvironment. Development.

[5] Kouros-Mehr H, Slorach EM, Sternlicht MD, Werb Z (2006). GATA-3 maintains the differentiation of the luminal cell fate in the mammary gland. Cell.

[6] Zaragoza R, Garcia-Trevijano ER, Lluch A, Ribas G, Vina JR (2015). Involvement of Different networks in mammary gland involution after the pregnancy/lactation cycle: Implications in breast cancer. IUBMB Life.

[7] Katz E, Streuli CH (2007). The extracellular matrix as an adhesion checkpoint for mammary epithelial function. Int J Biochem Cell Biol.

[8] Zuo Y, Berdeaux R, Frost JA (2014). The RhoGEF Net1 is required for normal mammary gland development. Mol Endocrinol.

[9] Radu M, Semenova G, Kosoff R, Chernoff J (2014). PAK signalling during the development and progression of cancer. Nat Rev Cancer.

[10] Kumar R, Gururaj AE, Barnes CJ (2006). p21-activated kinases in cancer. Nature Reviews Cancer.

[11] Rane CK, Minden A (2018). P21 activated kinase signaling in cancer. Semin Cancer Biol.

[12] Poonam R., Molli, D.-Q. L., Murray Brion, Suresh K. Rayala, Rakesh Kumar. PAK Signaling in Oncogenesis. 28 **28**, 2545–2555 (2010). 10.1038/onc.2009.119 PMC2731678 19465939

[13] Dart AE, Wells CM (2013). P21-activated kinase 4–not just one of the PAK. Eur J Cell Biol.

[14] Abo A (1998). PAK4, a novel effector for Cdc42Hs, is implicated in the reorganization of the actin cytoskeleton and in the formation of filopodia. Embo J.

[15] Li, Z. *et al.* Integrin-mediated Cell Attachment Induces a PAK4-dependent Feedback Loop Regulating Cell Adhesion through Modified Integrin αvβ5 Clustering and Turnover. *Molecular Biology of the Cell* **21**, 3317–3329 (2010). 10.1091/mbc.E10-03-0245 PMC2947468 20719960

[16] Callow MG (2002). Requirement for PAK4 in the anchorage-independent growth of human cancer cell lines. J Biol Chem.

[17] Li Z, *et al*. p21-activated kinase 4 phosphorylation of integrin beta5 Ser-759 and Ser-762 regulates cell migration. *J Biol Chem* **285**, 23699–23710 (2010). 10.1074/jbc.M110.123497 PMC2911335 20507994

[18] Zhang, H., Li, Z. & Viklund, EK. Strömblad S. P21-activated kinase 4 interacts with integrin alpha v beta 5 and regulates alpha v beta 5-mediated cell migration. *J Cell Biol* **158**, 1287–1297 (2002). 10.1083/jcb.200207008 PMC2173231 12356872

[19] Qu J (2003). PAK4 kinase is essential for embryonic viability and for proper neuronal development. Mol Cell Biol.

[20] Tian Y, Lei L, Cammarano M, Nekrasova T, Minden A (2009). Essential role for the Pak4 protein kinase in extraembryonic tissue development and vessel formation. Mech Dev.

[21] Tian Y, Lei L, Minden A (2011). A key role for Pak4 in proliferation and differentiation of neural progenitor cells. Dev Biol.

[22] Nekrasova TMA (2012). Role for p21-activated kinase PAK4 in development of the mammalian heart. Transgenic Res.

[23] Wagner KU, Ward MK, Davis T, Wiseman B, Hennighausen R (2001). L Spatial and temporal expression of the Cre gene under the control of the MMTV-LTR in different lines of transgenic mice. Transgenic Res.

[24] Schwenk F, Baron U, Rajewsky K (1995). A cre-transgenic mouse strain for the ubiquitous deletion of loxP-flanked gene segments including deletion in germ cells. Nucleic Acids Res.

[25] Wagner KU (1997). Cre-mediated gene deletion in the mammary gland. Nucleic Acids Res.

[26] Pylayeva Y (2009). Ras- and PI3K-dependent breast tumorigenesis in mice and humans requires focal adhesion kinase signaling. The Journal of clinical investigation.

[27] Laila Elsherif, M. O. *et al*. Potential Compensation among Group I PAK Members in Hindlimb Ischemia and Wound Healing. *PLoS One* **10** (2014). 10.1371/journal.pone.0112239 PMC4224450 25379771

[28] Boda BJL, Muller D (2008). Distinct, but compensatory roles of PAK1 and PAK3 in spine morphogenesis. Hippocampus.

[29] Zhuang T (2015). p21-activated kinase group II small compound inhibitor GNE-2861 perturbs estrogen receptor alpha signaling and restores tamoxifen-sensitivity in breast cancer cells. Oncotarget.

[30] Close D, Kesanakurti CC, Rajasekhar Maddirela D, Gujrati M, Rao JS (2012). Functional cooperativity by direct interaction between PAK4 and MMP-2 in the regulation of anoikis resistance, migration and invasion in glioma. Cell death Disease.

[31] Siu MK (2010). p21-Activated kinase-1 promotes aggressive phenotype, cell proliferation, and invasion in gestational trophoblastic disease. Am J Pathol.

[32] Talhouk RS, Chin JR, Unemori EN, Werb Z, Bissell MJ (1991). Proteinases of the mammary gland: developmental regulation *in vivo* and vectorial secretion in culture. Development.

[33] Correia ALMH, Chen EI, Schmitt FC, Bissell MJ (2013). The hemopexin domain of MMP3 is responsible for mammary epithelial invasion and morphogenesis through extracellular interaction with HSP90β. Genes Dev.

[34] Chernoff MLKaJ (2012). Mouse models of PAK function. Cell Logist.

[35] Zhao M (2017). Pdx1-Cre-driven conditional gene depletion suggests PAK4 as dispensable for mouse pancreas development. Sci Rep.

[36] Rui-An Wang RKV (2003). Essential functions of p21-activated kinase 1 in morphogenesis and differentiation of mammary glands. J Cell Biol.

[37] Li XMA (2003). Targeted disruption of the gene for the PAK5 kinase in mice. Mol Cell Bio.

[38] Furnari MA, Jobes ML, Nekrasova T, Minden A, Wagner GC (2014). Differential sensitivity of Pak5, Pak6, and Pak5/Pak6 double-knockout mice to the stimulant effects of amphetamine and exercise-induced alterations in body weight. Nutritional neuroscience.

[39] Costa TDF (2019). PAK4 suppresses RELB to prevent senescence-like growth arrest in breast cancer. Nat Commun.

[40] Guy CT, Cardiff RD, Muller WJ (1992). Induction of mammary tumors by expression of polyomavirus middle T oncogene: a transgenic mouse model for metastatic disease. Mol Cell Biol.

[41] Lin EY (2003). Progression to malignancy in the polyoma middle T oncoprotein mouse breast cancer model provides a reliable model for human diseases. Am J Pathol.

[42] Rennhack J, To B, Wermuth H, Andrechek ER. Mouse Models of Breast Cancer Share Amplification and Deletion Events with Human Breast Cancer. *J Mammary Gland Biol Neoplasia* **22**, 71–84 (2017). 10.1007/s10911-017-9374-y PMC5313323 28124185

[43] Robinson GW, Hennighausen L (2011). MMTV-Cre transgenes can adversely affect lactation: considerations for conditional gene deletion in mammary tissue. Anal Biochem.

[44] Sakamoto KSJ, Wagner K-U (2012). Generation of a Novel MMTV-tTA Transgenic Mouse Strain for the Targeted Expression of Genes in the Embryonic and Postnatal Mammary Gland. PLoS One.

[45] Cardiff RD, Miller CH, Munn RJ (2014). Manual hematoxylin and eosin staining of mouse tissue sections. Cold Spring Harb Protoc.

