# Supplementary material for: Normal mammary gland development after MMTV-Cre mediated conditional PAK4 gene depletion
Source: Sci Rep. 2019 Oct 8;9:14436. doi: 10.1038/s41598-019-50819-4 (PMC6783434; doi:10.1038/s41598-019-50819-4)
Supplement: Supplementary file 1 — Supplementary information [file 41598_2019_50819_MOESM1_ESM.pdf]

Supplementary Information

Normal mammary gland development after MMTV-Cre mediated conditional PAK4 gene depletion

Parisa Rabieifar[1#], Ting Zhuang[1,2#,] Tânia D. F. Costa[1], Miao Zhao[1,3], Staffan Strömblad[1*]

[1]Department of Biosciences and Nutrition, Karolinska Institutet, Stockholm, Sweden.

[2]Present address: Henan Collaborative Innovation Center of Molecular Diagnosis and Laboratory Medicine, School of Laboratory Medicine, Xinxiang Medical University, Xinxiang, Henan Province, P.R. China.

[3]Present address: Department of Immunology, Genetics and Pathology, Uppsala University, Uppsala, Sweden.

# These authors contributed equally to this work

*Correspondence to Staffan.Stromblad@ki.se

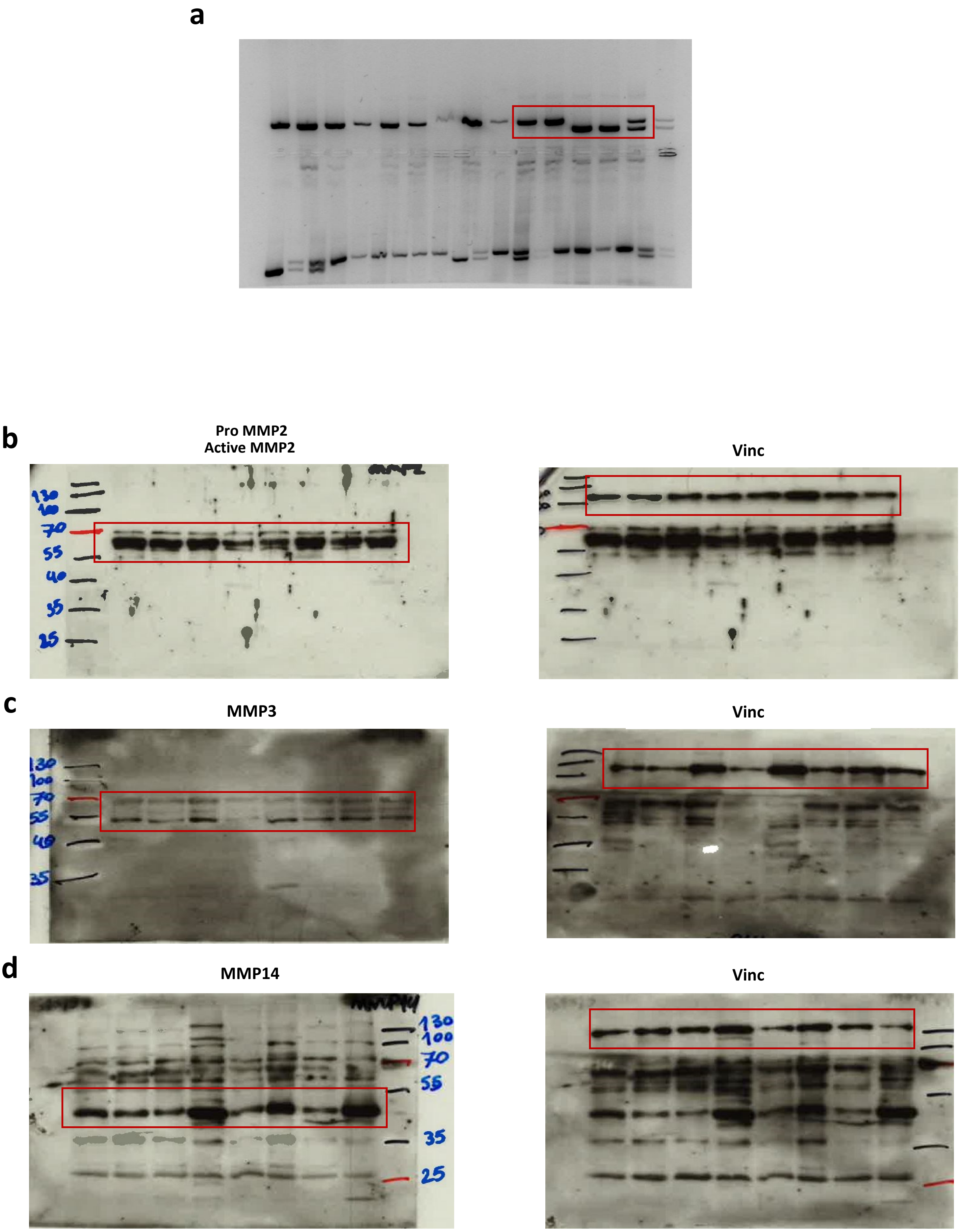

Supplementary Figure S1

(a) Uncropped gel image correspond to (Fig. 1 b)

(b-d) Uncropped western blot images correspond to (Fig. 3 c-d-e). Note that the size marker used in b and c (PageRuler) is different from the size marker used in d (PageRuler Plus).
